# Angiotensin converting enzyme gene polymorphism and influence of ACE inhibition

**DOI:** 10.4103/0971-4065.73433

**Published:** 2010-10

**Authors:** Q. Arman

**Affiliations:** University of Pennsylvania School of Medicine, Philadelphia, USA

Sir,

This letter is in reference to the article “Angiotensin converting enzyme gene polymorphism in type II diabetics with nephropathywhich suggests the association between the DD polymorphism and type II diabetes with nephropathy[1] in the Indian population. In the Caucasian population, the DD genotype has been associated with accelerated progression of other renal conditions too such as IgA nephropathy,[2] and FSGS. Studies on the I/D polymorphism also provides information on the variation of response to ACE inhibition in related studies. It has been seen in the Caucasian population that the DD genotype translated into more sensitivity to ramipril treatment.[3,4] However, subgroup analyses in these studies found that unlike in men, in women the response to ACE inhibition was fully independent of the underlying genotype. Hence, ramipril tended to provide renoprotection in men with DD genotype, but such a beneficial effect was not apparent in those with II or ID genotye. But women with the DD and the II or ID genotype had a response to ramipril completely independent of the genotype. Taking in account the high prevalence of diabetic nephropathy in the Indian population, it would be very interesting to know if there has been any evidence of variation in responses to ACE inhibition on the basis of angiotensin-converting enzyme gene polymorphism in type II diabetics with nephropathy in the Indian population. As such, an observation may markedly influence the scope of treatment in these patients.

## References

[1] Naresh V, Reddy A, Sivaramakrishna G, Sharma P, Vardhan RV, Kumar VS (2009). Angiotensin converting enzyme gene polymorphism in type II diabetics with nephropathy. Indian J Nephrol.

[2] Yoshida H, Kon V, Ichikawa I (1996). Polymorphisms of the renin-angiotensin system genes in progressive renal disease. Kidney Int.

[3] Yoshida H, Mitarai T, Kawamura T, Kitajima T, Miyazaki Y, Nagasawa R (1995). Role of the deletion polymorphism of the angiotensin converting enzyme gene in the progression and therapeutic responsiveness of IgA nephropathy. J Clin Invest.

[4] Schmidt S, Ritz E (1997). Genetics of the renin-angiotensin system and renal disease: A progress report. Curr Opin Nephrol Hypertens.

